# Reduction of calibration uncertainty due to mounting of three-axis accelerometers using the intrinsic properties model

**DOI:** 10.1088/1681-7575/abeccf

**Published:** 2021-04-13

**Authors:** Michael Gaitan, Iris Mariela López Bautista, Jon Geist

**Affiliations:** 1 National Institute of Standards and Technology Gaithersburg, MD 20899, United States of America; 2 Centro Nacional de Metrología Querétaro, C.P. 76246, México

**Keywords:** accelerometer, calibration, intrinsic

## Abstract

We show that the calibration of tri-axis accelerometers based on the device’s intrinsic properties alleviates the uncertainty due to mounting misalignment in comparison to the use of the sensitivity matrix. The intrinsic properties of a tri-axis accelerometer are based on a (*u*, v, *w*) coordinate system that represent the direction of maximum sensitivities of each of the three accelerometers (*U, V, W*) and are assumed not to be perfectly orthogonal to each other. The calibration procedure requires rotation of the device in the gravitational field around each of the Cartesian coordinate (*x, y, z*) axes. One component in driving down the uncertainty of laboratory comparisons and calibration repeats relates to misalignment in mounting the device onto the calibration instrument. We show that the uncertainty of the cross-axis terms of the sensitivity matrix is a dominating factor affecting uncertainty down to a 0.01° misalignment at a 100 μV noise level. The misalignment component can be exacerbated when calibrating modern microelectromechanical systems (MEMS)-based accelerometers, which are typically a few millimeters in dimension.

## 1. Introduction

Inertial measurement units (IMUs) have primarily been of key importance for guidance of vehicles such as ships, submarines, and aircraft. With the advent of microelectromechaical systems (MEMS) technology and the ability to miniaturize IMUs, their applications have expanded for use in electronic games, smart phones, drones, automobiles, and are continuing to grow in use, driving their manufacturing into many millions of units per year and pushing ever increasing performance needed for new applications such as autonomous vehicles and other systems requiring dead reckoning.

Requirements for testing and calibration of IMUs are dependent on their intended application. For applications where life and limb are not at stake, the manufacturers may use methods where a statistical sample of them are tested and calibrated while the rest only undergo a functional test. However, critical applications require rigorous testing and calibration of every device manufactured. The manufacturers, commercial calibration laboratories, test equipment manufacturers, and systems integrators have independently developed methods for testing and calibration where the calibration of tri-axis accelerometers is primarily based on their rotation in the gravitational field. However, there continues to be a metrological gap in this field of technology: comparison measurements to ensure the equivalence of measurements globally.

The consultative committee for acoustics, ultrasound and vibration (CCAUV) is one of 9 committees of the Bureau International ds Poids et Measures (BIPM), an ‘*intergovernmental organization through which Member States act together on matters related to measurement science and measurement standards*.’ CCAUV’s formal work is to conduct key comparisons of measurements related to acoustics (microphones and sound calibrators) and vibration (accelerometers). These key comparisons are conducted to ensure that measurements by the world’s National Metrology Institutes are known to be in agreement to within a stated degree of uncertainty that is determined through experiment by key comparisons.

The key comparisons currently supported by the CCAUV for accelerometers are associated with sinusoidal linear and angular vibration and shock based on the ISO 16063 series of standards on ‘Methods for the calibration of vibration and shock transducers’. However, the CCAUV’s key comparisons do not currently support tri-axial accelerometers. Towards this end there has been recent work by Prato *et al* [1] and others [2, 3] in developing vibration calibration methods for tri-axial accelerometers based on vibration measurements for the simultaneous determination of main and transverse sensitivities in the frequency domain. Prato *et al* employ an inclined mount for the accelerometer that is placed on a shaker. The accelerometer is rotated in a series of steps on the inclined mount to measure the response of the device at different angular positions resulting in the determination of its sensitivity matrix with the associated uncertainties. Umeda *et al* [2] employ a three-dimensional vibration generator and three laser interferometers.

Inertial calibrations, which are typically done by rotation of the device under test (DUT) in the gravitational field [4], lack rigorous development in the open literature and standards for laboratory comparisons and accreditation. One issue that we foresee is that MEMS-based devices, which are tiny in comparison to IMUs typically used for inertial guidance, can especially suffer from mounting misalignment of the DUT on the test instrument because of their small size. The sensitivity matrix is usually desired in a calibration, but we find that rotational misalignment will lead to added uncertainty in such a calibration for laboratory comparisons. To address the issue, we proposed the use of an intrinsic properties approach for laboratory comparisons [5].

The intrinsic properties of the device can be thought of as those properties of interest that can be assigned as inside the sensor package while the extrinsic properties can be thought of as those that affect the device response that are outside of the package. The intrinsic properties of a tri-axis accelerometer include the magnitude of each accelerometer’s sensitivity to acceleration, the degree of orthogonality of the accelerometers, and the effects of offset, time, and temperature. These properties can be considered independent of the alignment of the device on a test instrument.

In this paper we report on a detailed study of the effect of mounting misalignment on the intrinsic properties of the tri-axis accelerometer, specifically on the magnitude of the sensitivity of each of the accelerometers and their degree of orthogonality. We compare the intrinsic properties with the use of the sensitivity matrix. As will be shown, the intrinsic properties approach offers an advantage over the use of the sensitivity matrix for calibration laboratory comparisons of decreased uncertainty related to mounting misalignment.

## 2. Intrinsic properties model

**Figure 1.**
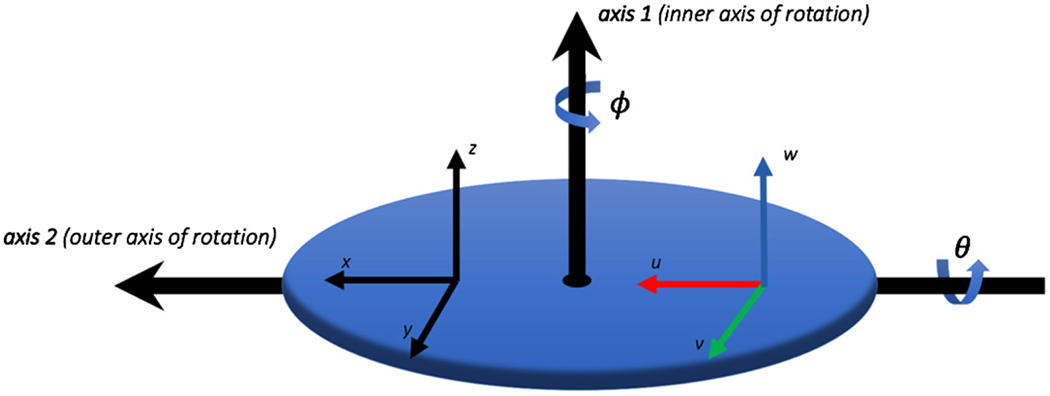
Depiction of the calibration instrument which is composed of a platter with orthogonal rotation axes axis 1 and axis 2 that have rotation angles *φ* and *θ*, respectively. An (*x, y, z*) Cartesian coordinate system is defined on the instrument platter with the *z* axis parallel to axis 1, the *x* axis parallel to axis 2 when (*φ* = 0), and axis 2 is parallel to the gravitational field when (*θ* = 0). The (*U*, *V*, *W*) accelerometer is placed on the rotation platter approximately aligned to the (*x*, *y*, *z*) coordinate system as shown.

The analysis given here is based on an earlier paper [5] devoted only to the theory of the intrinsic properties but employs a simplified notation. Consider a triaxial accelerometer DUT whose accelerometers are labeled (*U*, *V*, *W*). Assume that each of the accelerometers has a single maximum value of sensitivity along a unique axis defined by the unit vectors ($\hat{u}$, $\hat{\text{v}}$, $\hat{w}$) who’s directions are aligned to the maximum response of the (*U, V, W*) accelerometers, respectively. Furthermore, assume that the DUT has been mounted on a calibration system with its intrinsic (*u, v, w*) axes approximately aligned with the respective orthogonal (*x, y, z*) axes of the calibration system’s platter depicted in figure 1. The response of the DUT is related to the sensitivity matrix, the angle of rotation of axis 1 (*φ*) and axis 2 (*θ*), and the local acceleration due to gravity (*g*_loc_) by the equation:

(1) $$\left[\begin{array}{l} U(\theta ,\phi )\\ V(\theta ,\phi )\\ W(\theta ,\phi ) \end{array}\right]=g_{\text{loc}}\left[\begin{array}{ccc} s_{ux} & s_{uy} & s_{uz}\\ s_{vx} & s_{vy} & s_{vz}\\ s_{wx} & s_{wy} & s_{wz} \end{array}\right]\;\left[\begin{array}{c} \sin(\theta )\;\sin(\phi )\\ \sin(\theta )\;\cos(\phi )\\ \cos(\theta )\;\cos(\phi ) \end{array}\right]+\left[\begin{array}{c} O_{u}\\ O_{\upsilon }\\ O_{w} \end{array}\right],$$

and where the terms (*O*_*u*_, *O*_*v*_, *O*_*w*_) represent the DC offsets of the (*U, V, W*) accelerometers, respectively.

The components of the sensitivity matrix are represented in terms of an (*x, y, z*) Cartesian coordinate system with the *z* axis parallel to axis 1, and axis 1 and axis 2 are orthogonal to each other. Furthermore, the *x* axis is parallel to axis 2 and axis 1 is parallel to the gravitational field when the angles (*ϕ* = *θ* = 0). Equation (1) models the response of (*U, V, W*) accelerometers but also has equivalence to a three-axis device with perfectly orthogonal (*X*, *Y*, *Z*) accelerometers having main components of sensitivity *s_xx_*, *s_yy_*, and *s_zz_* in the direction of each of the axes and coupled with cross-axis components that respond to accelerations that are orthogonal to those main components,

(2) $$\left[\begin{array}{ccc} s_{xx} & s_{xy} & s_{xz}\\ s_{yx} & s_{yy} & s_{yz}\\ s_{zx} & s_{zy} & s_{zz} \end{array}\right]=\left[\begin{array}{ccc} s_{ux} & s_{uy} & s_{uz}\\ s_{vx} & s_{vy} & s_{vz}\\ s_{wx} & s_{wy} & s_{wz} \end{array}\right].$$

It is important to recognize that the sensitivity matrix components will vary as a function of any misalignment of the DUT to the axes of the calibration instrument.

We define the intrinsic properties of the DUT as nine parameters that are independent of the placement of the device on the calibration instrument. The first three intrinsic properties are the DC offsets (*O_u_, O_v_, O_w_*) of the (*U, V, W*) accelerometers, respectively. The second three intrinsic properties are the intrinsic sensitivities (*s_u_, s_v_, s_w_*) of the (*U, V, W*) accelerometers, which are defined as the response of each accelerometer to a unit acceleration along each of the (*u, v, w*) axes, given by:

(3) $$s_{u}=\sqrt{s_{ux}^{2}+s_{uy}^{2}+s_{uz}^{2}},$$

(4) $$s_{v}=\sqrt{s_{vx}^{2}+s_{vy}^{2}+s_{vz}^{2}},$$

(5) $$s_{w}=\sqrt{s_{wx}^{2}+s_{wy}^{2}+s_{wz}^{2}}.$$

The final three intrinsic properties are the angles between each of the (*U, V, W*) accelerometers:

(6) $$\varphi_{uv}=\cos^{-1}\left(\frac{s_{ux}s_{vx}+s_{uy}s_{vy}+s_{uz}s_{vz}}{s_{u}s_{v}}\right),$$

(7) $$\varphi_{vw}=\cos^{-1}\left(\frac{s_{vx}s_{wx}+s_{vy}s_{wy}+s_{vz}s_{wz}}{s_{v}s_{w}}\right),$$

(8) $$\varphi_{wu}=\cos^{-1}\left(\frac{s_{wx}s_{ux}+s_{wy}s_{uy}+s_{wz}s_{uz}}{s_{u}s_{w}}\right).$$

The first important point about equations (3)–(8) is that all of the quantities on the right-hand side of these equations can be calculated from the measured values of the sensitivity matrix. The second important point about equations (3)–(8) is that the six intrinsic quantities on the left-hand side of these equations are rigorously independent of alignment to the (*x*, *y*, *z*) coordinate system when three assumptions are satisfied.

The first assumption is that the (*x, y, z*) axes of the measurement instrument are orthogonal and in its initial condition (*φ* = *θ* = 0) the *z* axis is aligned to the direction of the gravitational field. The second assumption is that the response of the (*U*, *V*, *W*) accelerometers is a linear function of the acceleration along the (*u*, *v, w*) axes. The third assumption is that the intrinsic properties are independent of time and the local values of environmental parameters such as temperature, humidity, pressure, and magnetic field are fixed and independent of them, however, these parameters can be included in the analysis if desired. The extent to which these assumptions are true will determine the extent to which equations (3)–(8) are satisfied. It is important to understand that the (*u, v, w*) axes do not need to be mutually perpendicular, but large deviations from perpendicularity may increase the uncertainty of the result.

## 3. Calibration protocol and data analysis

**Figure 2.**
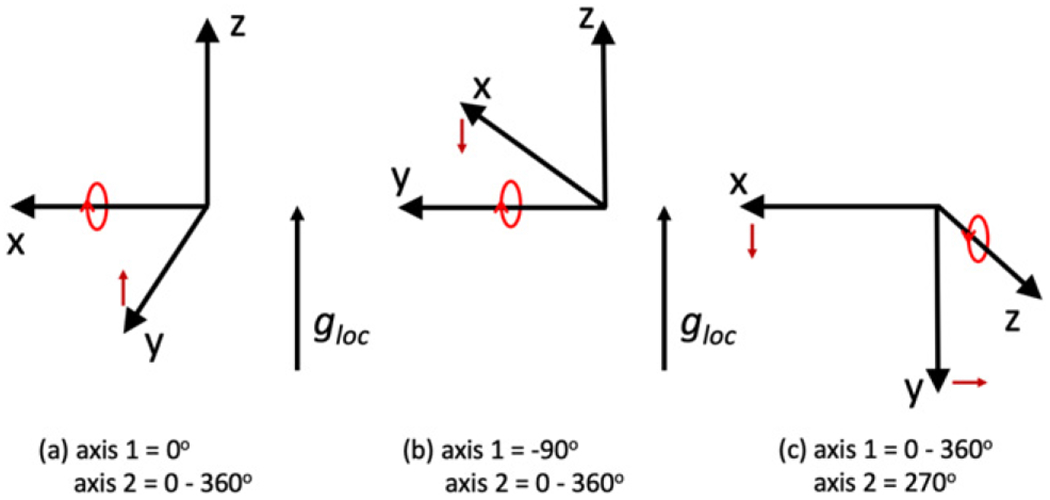
The calibration protocol to determine the DUT’s intrinsic properties requires rotation around each of the (*x*, *y*, *z*) axes with the axis of rotation positioned perpendicular to the direction of gravity and depicted here in three parts: (a) axis 2 of the measuring instrument is rotated in steps around 360° with axis 1 set to 0°, (b) axis 2 is rotated in steps around 360° with axis 1 set to −90°, and (c) axis 1 is rotated in steps around 360° with axis 2 set to 90°. The term *g*_loc_ represents the local gravitational acceleration pointing in the direction that is detected by the accelerometer.

The orientation of the (*x*, *y*, *z*) coordinate system of the measuring instrument and (*u*, *v*, *w*) coordinate system of the DUT is depicted in figure 1, which depicts the two-axes of rotation of the measuring instrument, labeled as axis 1 and axis 2. The accuracy of the calibration requires knowledge that the measuring instrument has been calibrated and characterized so that each of the (*x, y, z*) axes are orthogonal and the degree of rotation of the axes and the local acceleration due to gravity are known to a stated uncertainty. The calibration protocol requires rotation around each of the (*x, y, z*) axes with the axis of rotation positioned to be perpendicular to the direction of gravity, depicted in figure 2 as rotation conditions (a)–(c). The DUT has (*U*, *V*, *W*) accelerometers with the axes (*u*, *v*, *w*) placed on the measuring instrument in approximate congruence with the instrument’s (*x, y, z*) axes.

In each of the three rotation conditions shown in figures 2(a)–(c), the DUT is rotated in chosen steps by the operator to complete a full 360° rotation and the responses of the (*U, V*, *W*) accelerometers are recorded at each rotation angle. Each of the responses could be a single reading or an average and standard deviation of a time series of readings at each rotation angle. This will result in a list of measurements for the responses of (*U, V, W*) that are a function of the angle of rotation, *α*. For example, for rotation condition (a) around the *x* axis, for readings *i* = 1,…, *N* of the rotation angle *α* we have a list of readings represented by the *i*^th^ reading,

(9) $$\left[\begin{array}{l} U^{x}\left(\alpha_{i}\right)\\ V^{x}\left(\alpha_{i}\right)\\ W^{x}\left(\alpha_{i}\right) \end{array}\right]=g_{loc}\left[\begin{array}{ccc} s_{ux}^{x} & s_{uy}^{x} & s_{uz}^{x}\\ s_{vx}^{x} & s_{vy}^{x} & s_{vz}^{x}\\ s_{wx}^{x} & s_{wy}^{x} & s_{uz}^{x} \end{array}\right]\;\left[\begin{array}{c} 0\\ \sin\left(\alpha_{i}\right)\\ \cos\left(\alpha_{i}\right) \end{array}\right]+\left[\begin{array}{c} O_{u}^{x}\\ O_{v}^{x}\\ O_{w}^{x} \end{array}\right]\;=\left[\begin{array}{c} A_{uy}^{x}\;\sin\left(\alpha_{i}\right)+B_{uz}^{x}\;\cos\left(\alpha_{i}\right)\\ A_{vy}^{x}\;\sin\left(\alpha_{i}\right)+B_{vz}^{x}\;\cos\left(\alpha_{i}\right)\\ A_{wy}^{x}\;\sin\left(\alpha_{i}\right)+B_{wz}^{x}\;\cos\left(\alpha_{i}\right) \end{array}\right]+\left[\begin{array}{c} O_{u}^{x}\\ O_{v}^{x}\\ O_{w}^{x} \end{array}\right],$$

where *U*^*x*^ (*α_i_*) is the reading from the *U* accelerometer at the angle of the rotation *α*_*i*_ of the *x* axis. The matrix components *s*^*x*^_*jk*_ make up the sensitivity matrix with the subscript *j* representing the *U, V*, or *W* accelerometers and the subscript *k* representing the response of the *x, y*, or *z* component of that accelerometer. The constant *g*_loc_ is the local acceleration due to gravity used in ISO Standard 16063-42 [6] and which has been measured at a reference location at the National Institute of Standards and Technology’s (NIST) Gaithersburg campus to be 9.801 018 m s^−2^ ± 5 × 10^−6^ m s^−2^ [7]. A correction for the difference in elevation *h* between the measurement instrument and the reference location, which is given by 3 × 10^−7^ h s^−2^, is negligible for the results reported here. The units for *A* and *B* are in the units of the measured accelerometer response per *g*_loc_. Since the rotation is around the *x* axis, the response for the *x* components of each of the (*U, V, W*) accelerometers is zero, while the *y* and *z* components follow a sine and cosine functionality.

The same notation is followed giving a similar list of equations for rotation around the *y* axis (b) and rotation around the *z* axis (c). For simplicity the same step angle can be used for each of the three rotations, hence we use the same variable *α*_*i*_ resulting in,

(10) $$\left[\begin{array}{c} U^{y}\left(\alpha_{i}\right)\\ V^{y}\left(\alpha_{i}\right)\\ W^{y}\left(\alpha_{i}\right) \end{array}\right]=g_{\text{loc}}\left[\begin{array}{ccc} s_{ux}^{y} & s_{uy}^{y} & s_{uz}^{y}\\ s_{vx}^{y} & s_{vy}^{y} & s_{vz}^{y}\\ s_{wx}^{y} & s_{wy}^{y} & s_{wz}^{y} \end{array}\right]\;\left[\begin{array}{c} -\sin\left(\alpha_{i}\right)\\ 0\\ \cos\left(\alpha_{i}\right) \end{array}\right]+\left[\begin{array}{c} O_{u}^{y}\\ O_{v}^{y}\\ O_{w}^{y} \end{array}\right]\;=\left[\begin{array}{c} -A_{ux}^{y}\;\sin\left(\alpha_{i}\right)+B_{uz}^{y}\;\cos\left(\alpha_{i}\right)\\ -A_{vx}^{y}\;\sin\left(\alpha_{i}\right)+B_{vz}^{y}\;\cos\left(\alpha_{i}\right)\\ -A_{wx}^{y}\;\sin\left(\alpha_{i}\right)+B_{wz}^{y}\;\cos\left(\alpha_{i}\right) \end{array}\right]+\left[\begin{array}{c} O_{u}^{y}\\ O_{v}^{y}\\ O_{w}^{y} \end{array}\right],$$

(11) $$\left[\begin{array}{c} U^{z}\left(\alpha_{i}\right)\\ V^{z}\left(\alpha_{i}\right)\\ W^{z}\left(\alpha_{i}\right) \end{array}\right]=g_{\mathrm{loc}}\left[\begin{array}{ccc} s_{ux}^{z} & s_{uy}^{z} & s_{uz}^{z}\\ s_{vx}^{z} & s_{vy}^{z} & s_{vz}^{z}\\ s_{wx}^{z} & s_{wy}^{z} & s_{wz}^{z} \end{array}\right]\;\left[\begin{array}{c} -\sin\left(\alpha_{i}\right)\\ -\cos\left(\alpha_{i}\right)\\ 0 \end{array}\right]+\left[\begin{array}{c} O_{u}^{y}\\ O_{v}^{y}\\ O_{w}^{y} \end{array}\right]\;=\left[\begin{array}{c} -A_{ux}^{z}\;\sin\left(\alpha_{i}\right)-B_{uy}^{z}\;\cos\left(\alpha_{i}\right)\\ -A_{vx}^{z}\;\sin\left(\alpha_{i}\right)-B_{vy}^{z}\;\cos\left(\alpha_{i}\right)\\ -A_{wx}^{z}\;\sin\left(\alpha_{i}\right)-B_{wy}^{z}\;\cos\left(\alpha_{i}\right) \end{array}\right]+\left[\begin{array}{c} O_{u}^{z}\\ O_{v}^{z}\\ O_{w}^{z} \end{array}\right],$$

where $U^{k}\left(\alpha_{i}\right),V^{k}\left(\alpha_{i}\right),$ and $W^{k}\left(\alpha_{i}\right)$ represent the voltage readings from the (*U*, *V*, *W*) accelerometers as a function of the rotation angle *α*_*i*_ and the superscripts represent a rotation around the *x, y*, or *z* axes.

It should be noted that the + or − prefactors for the *A_jk_* and *B_jk_* constants in equations (9)–(11) are correct for the alignment and rotations defined in figure 2 and as expressed in equation (1) but could differ if the alignment and rotations of the particular measuring instrument are different.

The offsets of the accelerometers, (*O*_*u*_, *O*_*v*_, *O*_*w*_) can include both a temperature coefficient of the offset and a time dependent drift coefficient of the offset. These can be included in the analysis by respectively substituting them in equations (9)–(11) by the expressions,

(12) $$\left[\begin{array}{c} O_{u}^{x}+C_{u}^{x}\left(t-t_{0}\right)+D_{u}^{x}\left(T-T_{0}\right)\\ O_{v}^{x}+C_{v}^{x}\left(t-t_{0}\right)+D_{v}^{x}\left(T-T_{0}\right)\\ O_{w}^{x}+C_{w}^{x}\left(t-t_{0}\right)+D_{w}^{x}\left(T-T_{0}\right) \end{array}\right]\;\left[\begin{array}{c} O_{u}^{y}+C_{u}^{y}\left(t-t_{0}\right)+D_{u}^{y}\left(T-T_{0}\right)\\ O_{v}^{y}+C_{v}^{y}\left(t-t_{0}\right)+D_{v}^{y}\left(T-T_{0}\right)\\ O_{w}^{y}+C_{w}^{y}\left(t-t_{0}\right)+D_{w}^{y}\left(T-T_{0}\right) \end{array}\right]\;\left[\begin{array}{c} O_{u}^{z}+C_{u}^{z}\left(t-t_{0}\right)+D_{u}^{z}\left(T-T_{0}\right)\\ O_{v}^{z}+C_{v}^{z}\left(t-t_{0}\right)+D_{v}^{z}\left(T-T_{0}\right)\\ O_{w}^{z}+C_{w}^{z}\left(t-t_{0}\right)+D_{w}^{z}\left(T-T_{0}\right) \end{array}\right],$$

where $C_{k}^{x}$ represents the time coefficient of the offset for the rotation around the *x* axis, (*t* – *t*_0_) represents the time passed since the start of the measurement *t*_0_, $D_{k}^{x}$ represents the temperature coefficient of the offset, and (*T* – *T*_0_) represents the temperature difference from a reference temperature *T*_0_.

We differentiate the measured sensitivity matrix coefficients with the superscript *x, y*, or *z* in equations (9)–(12) because calculations of duplicate values of the same quantity derived from fits with different rotations should be similar but will not necessarily give exactly the same results for each of the three rotations. The zero values in the sine/cosine vectors appear because there is no excitation in the direction parallel to the axis of rotation.

The series of measurements for each of the three rotations can be fit to sine using a least squares analysis of the sort,

(13) $$\left[\begin{array}{c} U\left(\alpha_{i}\right)\\ V\left(\alpha_{i}\right)\\ W\left(\alpha_{i}\right) \end{array}\right]=\left[\begin{array}{c} \pm A_{u}\;\sin\left(\alpha_{i}\right)\pm B_{u}\;\cos\left(\alpha_{i}\right)+O_{u}+C_{u}\left(t-t_{0}\right)+D_{u}\left(T-T_{0}\right)\\ \pm A_{v}\;\sin\left(\alpha_{i}\right)\pm B_{v}\;\cos\left(\alpha_{i}\right)+O_{v}+C_{v}\left(t-t_{0}\right)+D_{v}\left(T-T_{0}\right)\\ \pm A_{w}\;\sin\left(\alpha_{i}\right)\pm B_{w}\;\cos\left(\alpha_{i}\right)+O_{w}+C_{w}\left(t-t_{0}\right)+D_{w}\left(T-T_{0}\right) \end{array}\right].$$

Fitting this set of equations for each of the rotations will result in 3 matrices with values for *A* and *B* to yield,

(14) $$\left[\begin{array}{ccc} 0 & A_{uy}^{x} & B_{uz}^{x}\\ 0 & A_{vy}^{x} & B_{vz}^{x}\\ 0 & A_{wy}^{x} & B_{wz}^{x} \end{array}\right],$$

(15) $$\left[\begin{array}{ccc} A_{ux}^{y} & 0 & B_{ux}^{y}\\ A_{vx}^{y} & 0 & B_{vx}^{y}\\ A_{wx}^{y} & 0 & B_{wx}^{y} \end{array}\right],$$

(16) $$\left[\begin{array}{ccc} A_{ux}^{z} & B_{ux}^{z} & 0\\ A_{vx}^{z} & B_{ux}^{z} & 0\\ A_{wx}^{z} & B_{ux}^{z} & 0 \end{array}\right],$$

and three matrices for the offsets of the sort,

(17) $$\left[\begin{array}{ccc} O_{u}^{x} & C_{u}^{x} & D_{x}^{u}\\ O_{v}^{x} & C_{v}^{x} & D_{v}^{u}\\ O_{w}^{x} & C_{w}^{x} & D_{w}^{u} \end{array}\right],$$

(18) $$\left[\begin{array}{ccc} O_{u}^{y} & C_{u}^{y} & D_{x}^{y}\\ O_{v}^{y} & C_{v}^{y} & D_{v}^{y}\\ O_{w}^{y} & C_{w}^{y} & D_{w}^{y} \end{array}\right],$$

(19) $$\left[\begin{array}{ccc} O_{u}^{z} & C_{u}^{z} & D_{x}^{z}\\ O_{v}^{z} & C_{v}^{z} & D_{v}^{z}\\ O_{w}^{z} & C_{w}^{z} & D_{w}^{z} \end{array}\right].$$

The sensitivity matrix and offset vector can be calculated by taking the average of the values in equations (14)–(19) by the relationship,

(20) $$\left[\begin{array}{ccc} s_{ux} & s_{uy} & s_{uz}\\ s_{vx} & s_{vy} & s_{vz}\\ s_{wx} & s_{wy} & s_{wz} \end{array}\right]=\frac{1}{2g_{\mathrm{loc}}}\left[\begin{array}{ccc} \left(A_{ux}^{y}+A_{ux}^{z}\right) & \left(A_{uy}^{x}+B_{uy}^{z}\right) & \left(B_{uz}^{x}+B_{uz}^{y}\right)\\ \left(A_{vx}^{y}+A_{vx}^{z}\right) & \left(A_{vy}^{x}+B_{vy}^{z}\right) & \left(B_{vz}^{x}+B_{vz}^{y}\right)\\ \left(A_{wx}^{y}+A_{wx}^{z}\right) & \left(A_{wy}^{x}+B_{wy}^{z}\right) & \left(B_{wz}^{x}+B_{wz}^{y}\right) \end{array}\right],$$

and

(21) $$\left[\begin{array}{ccc} O_{u} & C_{u} & D_{u}\\ O_{v} & C_{v} & D_{v}\\ O_{w} & C_{w} & D_{w} \end{array}\right]=\frac{1}{3}\left[\begin{array}{ccc} \left(O_{u}^{x}+O_{u}^{y}+O_{u}^{z}\right) & \left(C_{u}^{x}+C_{u}^{y}+C_{u}^{\tau }\right) & \left(D_{u}^{\tau }+D_{u}^{y}+D_{u}^{z}\right)\\ \left(O_{v}^{x}+O_{v}^{y}+O_{v}^{z}\right) & \left(C_{v}^{x}+C_{v}^{y}+C_{v}^{z}\right) & \left(D_{v}^{x}+D_{v}^{y}+D_{v}^{z}\right)\\ \left(O_{w}^{x}+O_{w}^{y}+O_{w}^{z}\right) & \left(C_{w}^{x}+C_{w}^{y}+C_{w}^{z}\right) & \left(D_{w}^{x}+D_{w}^{y}+D_{w}^{z}\right) \end{array}\right].$$

Finally, the intrinsic properties can be calculated from equations (20) and (21) using equations (3)–(8).

## 4. Experimental design

The objective of this experiment was to investigate the effect of mounting misalignment of the DUT on the calibration of its intrinsic properties and to compare it to the sensitivity matrix. In this section we present a description of the test equipment used, the description and calibration of the DUT used in this study, the procedure that was used to simulate the effect of mounting misalignment, and the experimental results observed when applying the calibration procedure with a purposely applied rotational misalignment as well as constrained quasi-random misalignments.

### 4.1. Calibration equipment

**Figure 3.**
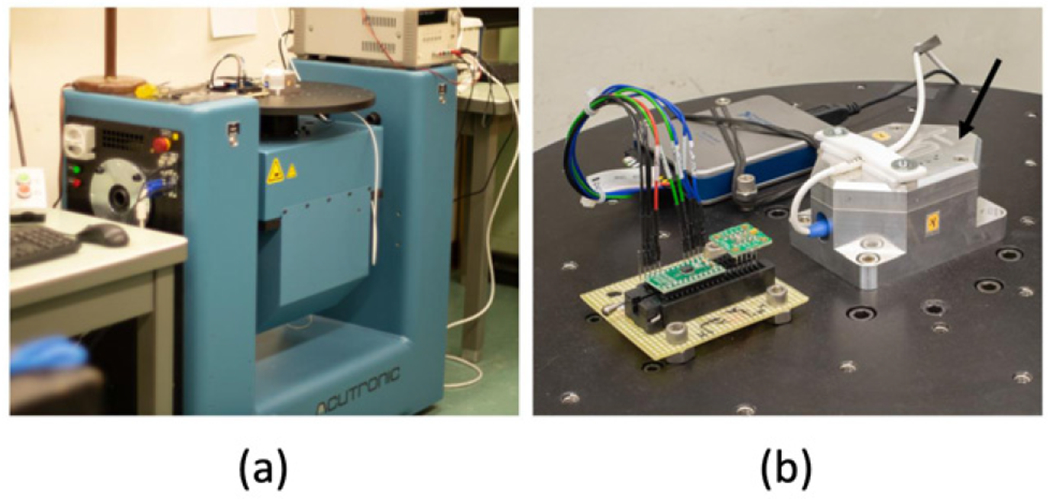
(a) Photograph of the AC216 two-axis rotation and rate table manufactured by acutronic and (b) a closeup view of the mounted Dytran 7503D1 three-axis accelerometer (DUT) used in the study. The arrow in (b) points to the placement of the DUT, which is mounted inside an aluminum chassis and bolted onto the platter of the test equipment. Also pictured in (b) to the left of the DUT are a pair of digital MEMS-based accelerometers.

The calibration equipment used in the experiment, shown in figure 3(a), was a commercially available AC216-CR two-axis rotation table manufactured by Acutronic [8], with a manufacturer’s specified rotational accuracy of ±0.001°. The test equipment’s platter rotates around two axes as depicted in figure 1, labeled axis 1 and axis 2. The equipment was aligned during installation so that the surface of the platter faced upwards (perpendicular to the direction of gravity) using a precision engineering spirit level with sensitivity of 0.01 mm m^−1^ (0.0006°).

The procedure that we used to further characterize the performance of the instrument is lengthy and will be reported in a publication to follow but the specifications described here are adequate to support the experimental observations.

### 4.2. Description and calibration of the triaxial accelerometer (DUT)

The accelerometer used in this experiment, Dytran 7503D1 triaxial DC accelerometer shown in figure 3(b), is housed inside an aluminum casing and bolted onto the rotation stage. We currently use this accelerometer as our check standard. It produces an analog output voltage for which the manufacturer specifies a ±2 *g*_*n*_ input range (±19.6 m s^−2^), a 2000 mV/*g_n_* (±200 mV m^−1^ s^−2^) sensitivity^3^, a maximum cross-axis sensitivity of 3%, and a ±50 mV maximum offset [9]. The voltage readings from the accelerometer were measured using a National Instruments 9239 analog input module for which the manufacturer specifies a ±0.03% gain error and ±0.008% offset error, and interfaced to a computer running a LabVIEW program to control the angular rotation of axis 1 and axis 2, and to tabulate the angular positions and voltage readings.

Following the calibration protocol illustrated in figure 2, the accelerometer was rotated around each of its (*x, y, z*) axes in 5° steps:

- Axis 2 of the measuring instrument is rotated in 5° steps around 360° with axis 1 set to 0°
- Axis 2 is rotated in 5° steps around 360° with axis 1 set to −90°
- Axis 1 is rotated in 5° steps around 360° with axis 2 set to 270°

And resulted in 72 readings for each of the three rotations at rotation angles {0°, 5°, 10°, 15°, …, 355°}.

**Figure 4.**
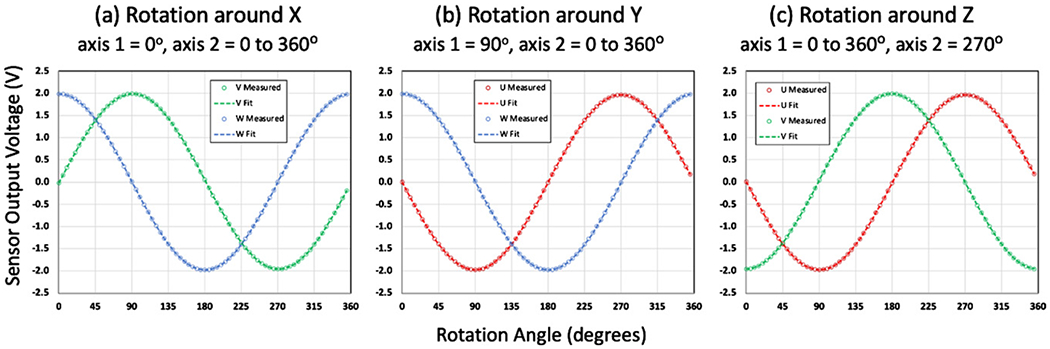
Measurement and sine fitting results for rotation around (a) the *x* axis, (b) the *y* axis, and (c) the *z* axis following the protocol depicted in figure 2. The results for the *U* accelerometer are depicted in red, the *V* accelerometer in green, and the *W* accelerometer in blue.

**Figure 5.**
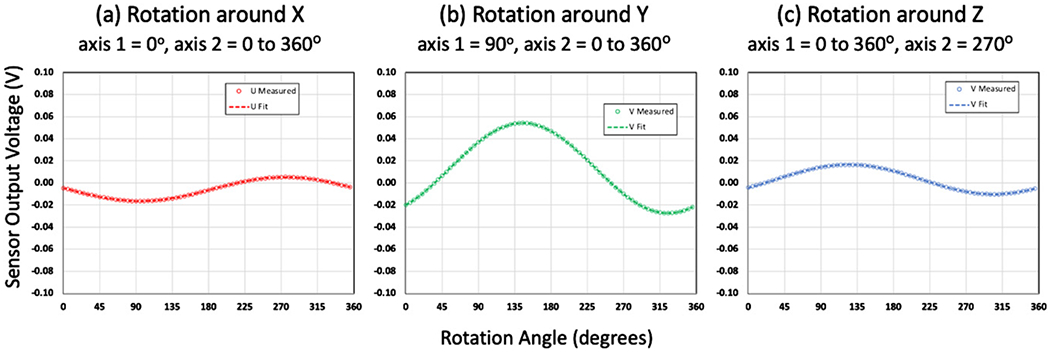
Measurement and sine fitting results for (a) *U* accelerometer readings for rotation around the *x* axis, (b) *V* accelerometer readings for rotation around the *y* axis, and (c) *W* accelerometer readings for rotation around the *z* axis. These are plotted separately from figure 4 since their magnitude is much smaller.

The accelerometer readings for the calibration are plotted in figure 4 for accelerometers perpendicular to each axis of rotation and figure 5 for accelerometers parallel to each axis of rotation. In these calculations the variables *C* and *D* were not included since the measurements were made over a short period of time at a constant ambient temperature of 25 °C. The measurements were sine fitted with Microsoft Excel using the LINEST function following the form of equation (13) to yield the values of *A*, *B*, and *O* described in equations (14)–(19) listed in table 1.

Using the values from table 1, the sensitivity matrix in the units of V ms^−2^ are calculated following equation (19) to yield,

(22) $$\left[\begin{array}{ccc} s_{ux} & s_{uy} & s_{uz}\\ s_{vx} & s_{vy} & s_{vz}\\ s_{wx} & s_{wy} & s_{wz} \end{array}\right]=\frac{1}{g_{\text{loc}}}\left[\begin{array}{ccc} 1.97351 & -0.01063 & 0.00058\\ -0.02368 & 1.97595 & -0.03315\\ -0.01087 & 0.00731 & 1.98421 \end{array}\right]\;=\left[\begin{array}{ccc} 0.201358 & -0.001085 & 0.000059\\ -0.002416 & 0.201607 & -0.003382\\ -0.001109 & 0.000746 & 0.202449 \end{array}\right].$$

The values for the offset voltages are determined from equation (20) to yield the values in mV are,

(23) $$\left[\begin{array}{c} O_{u}\\ O_{v}\\ O_{w} \end{array}\right]=\left[\begin{array}{c} -5.146\\ 13.879\\ 2.966 \end{array}\right].$$

The intrinsic properties of the accelerometer are determined following equations (3)–(5) to yield the values for the sensitivities of the (*U*, *V*, *W*) accelerometers in the units of V ms^−2^,

(24) $$s_{u}=\sqrt{s_{ux}^{2}+s_{uy}^{2}+s_{uz}^{2}}=0.201361\;s_{v}=\sqrt{s_{vx}^{2}+s_{vy}^{2}+s_{vz}^{2}}=0.201649,\;s_{w}=\sqrt{s_{wx}^{2}+s_{wy}^{2}+s_{wz}^{2}}=0.202454$$

and using equations (6)–(8) to determine the angles between the (*U*, *V*, *W*) accelerometers in degrees,

(25) $$\varphi_{uv}=90.995\;\varphi_{vw}=90.746\;\varphi_{wu}=90.298.$$

### 4.3. Calibration uncertainties

Least squares sine fitting using a fitting function such as LINEST in Microsoft Excel will yield standard error values for the fitting coefficients that can in turn be used to estimate measurement uncertainties. Fitting a function of the form,

(26) f(α)=Asin(α)+Bcos(α)+O,

will yield the coefficients *A*, *B*, and *O* and their standard errors which we denote as SE {*A*}, SE {*B*}, and SE {*O*}, respectively. The standard errors resulting from sine fitting can then be used in accordance with equations (13)–(21) to estimate the uncertainties of the sensitivity matrix and the intrinsic properties. From equation (21),

(27) $$s_{ux}=\frac{1}{2g_{loc}}\left(A_{ux}^{y}+A_{ux}^{z}\right),$$

and its associated standard error is described as,

(28) $$\text{SE}\left\{s_{ux}\right\}=\frac{1}{g_{\mathrm{loc}}}\text{SE}\left\{A_{ux}\right\}\;=\frac{1}{g_{\mathrm{loc}}}\sqrt{\frac{\text{SE}{\left\{A_{ux}^{y}\right\}}^{2}+\text{SE}{\left\{A_{ux}^{z}\right\}}^{2}}{2}}.$$

The standard errors for the entire sensitivity matrix can be determined following the same form in equation (28) to yield,

(29) $$\left[\begin{array}{ccc} \text{SE}\left\{A_{ux}\right\} & \text{SE}\left\{A_{uy}\right\} & \text{SE}\left\{A_{uz}\right\}\\ \text{SE}\left\{A_{vx}\right\} & \text{SE}\left\{A_{vy}\right\} & \text{SE}\left\{A_{vz}\right\}\\ \text{SE}\left\{A_{wx}\right\} & \text{SE}\left\{A_{wy}\right\} & \text{SE}\left\{A_{wz}\right\} \end{array}\right]=\frac{1}{\sqrt{2}}\left[\begin{array}{ccc} \sqrt{\mathrm{SE}{\left\{A_{ux}^{y}\right\}}^{2}+\mathrm{SE}{\left\{A_{ux}^{z}\right\}}^{2}} & \sqrt{\mathrm{SE}{\left\{A_{uy}^{x}\right\}}^{2}+\mathrm{SE}{\left\{B_{uy}^{z}\right\}}^{2}} & \sqrt{\mathrm{SE}{\left\{B_{uz}^{x}\right\}}^{2}+\mathrm{SE}{\left\{B_{uz}^{y}\right\}}^{2}}\\ \sqrt{\mathrm{SE}{\left\{A_{vx}^{y}\right\}}^{2}+\mathrm{SE}{\left\{A_{vx}^{z}\right\}}^{2}} & \sqrt{\mathrm{SE}{\left\{A_{iy}^{x}\right\}}^{2}+\mathrm{SE}{\left\{B_{vy}^{z}\right\}}^{2}} & \sqrt{\mathrm{SE}{\left\{B_{vz}^{x}\right\}}^{2}+\mathrm{SE}{\left\{B_{vz}^{y}\right\}}^{2}}\\ \sqrt{\mathrm{SE}{\left\{A_{wx}^{y}\right\}}^{2}+\mathrm{SE}{\left\{A_{wx}^{z}\right\}}^{2}} & \sqrt{\mathrm{SE}{\left\{A_{wy}^{x}\right\}}^{2}+\mathrm{SE}{\left\{B_{wy}^{z}\right\}}^{2}} & \sqrt{\mathrm{SE}{\left\{B_{wz}^{x}\right\}}^{2}+\mathrm{SE}{\left\{B_{wz}^{y}\right\}}^{2}} \end{array}\right].$$

The magnitudes of the intrinsic properties follow the form of equation (24),

(30) $$s_{u}=\sqrt{s_{ux}^{2}+s_{uy}^{2}+s_{uz}^{2}},$$

resulting in the equations for the standard error for the intrinsic properties of,

(31) $$\mathrm{SE}\left\{s_{u}\right\}=\sqrt{\frac{{\left(s_{ux}\mathrm{SE}\left\{s_{ux}\right\}\right)}^{2}+{\left(s_{uy}\mathrm{SE}\left\{s_{uy}\right\}\right)}^{2}+{\left(s_{uz}\mathrm{SE}\left\{s_{uz}\right\}\right)}^{2}}{s_{u}^{2}}}\;\mathrm{SE}\left\{s_{v}\right\}=\sqrt{\frac{{\left(s_{vx}\mathrm{SE}\left\{s_{vx}\right\}\right)}^{2}+{\left(s_{vy}\mathrm{SE}\left\{s_{vy}\right\}\right)}^{2}+{\left(s_{vz}\mathrm{SE}\left\{s_{vz}\right\}\right)}^{2}}{s_{v}^{2}}}.\;\mathrm{SE}\left\{s_{w}\right\}=\sqrt{\frac{{\left(s_{ux}\mathrm{SE}\left\{s_{wx}\right\}\right)}^{2}+{\left(s_{wy}\mathrm{SE}\left\{s_{wy}\right\}\right)}^{2}+{\left(s_{wz}\mathrm{SE}\left\{s_{wz}\right\}\right)}^{2}}{s_{w}^{2}}}$$

The intrinsic angles follow the form of equation (6),

(32) $$s_{u}s_{v}\;\cos\left(\varphi_{uv}\right)=s_{ux}s_{vx}+s_{uy}s_{vv}+s_{uz}s_{vz},$$

resulting in a standard error for the intrinsic angles of,

(33) $$\mathrm{SE}\left\{\varphi_{uv}\right\}\approx \sqrt{\frac{{\left(\mathrm{SE}\left\{s_{ux}\right\}s_{vx}\right)}^{2}+{\left(\mathrm{SE}\left\{s_{vx}\right\}s_{ux}\right)}^{2}+{\left(\mathrm{SE}\left\{s_{uy}\right\}s_{vy}\right)}^{2}+{\left(\mathrm{SE}\left\{s_{vy}\right\}s_{uy}\right)}^{2}+{\left(\mathrm{SE}\left\{s_{uz}\right\}s_{vz}\right)}^{2}+{\left(\mathrm{SE}\left\{s_{vz}\right\}s_{uz}\right)}^{2}}{{\left(s_{u}s_{v}\right)}^{2}}}\;\mathrm{SE}\left\{\varphi_{vw}\right\}\approx \sqrt{\frac{{\left(\mathrm{SE}\left\{s_{vx}\right\}s_{wx}\right)}^{2}+{\left(\mathrm{SE}\left\{s_{wx}\right\}s_{vx}\right)}^{2}+{\left(\mathrm{SE}\left\{s_{vw}\right\}s_{wy}\right)}^{2}+{\left(\mathrm{SE}\left\{s_{wy}\right\}s_{vy}\right)}^{2}+{\left(\mathrm{SE}\left\{s_{vz}\right\}s_{wz}\right)}^{2}+{\left(\mathrm{SE}\left\{s_{wz}\right\}s_{vz}\right)}^{2}}{{\left(s_{v}s_{w}\right)}^{2}}},\;\mathrm{SE}\left\{\varphi_{wu}\right\}\approx \sqrt{\frac{{\left(\mathrm{SE}\left\{s_{ux}\right\}s_{wx}\right)}^{2}+{\left(\mathrm{SE}\left\{s_{wx}\right\}s_{ux}\right)}^{2}+{\left(\mathrm{SE}\left\{s_{uy}\right\}s_{wy}\right)}^{2}+{\left(\mathrm{SE}\left\{s_{wy}\right\}s_{uy}\right)}^{2}+{\left(\mathrm{SE}\left\{s_{uz}\right\}s_{wz}\right)}^{2}+{\left(\mathrm{SE}\left\{s_{wz}\right\}s_{uz}\right)}^{2}}{{\left(s_{u}s_{w}\right)}^{2}}}$$

where sin(*φ*) ≈ 1 since the deviation of these angles from 90° is small.

### 4.4. Modeling the effect of measurement noise and misalignment

The effects of misalignment and measurement noise on the calibration and calibration uncertainties can be modeled using an analysis based on equation (2) given a sensitivity matrix that is measured with no misalignment. In this model, the plane of *u*–*v* accelerometers is assumed to be parallel to the *x*–*y* plane of the measuring instrument and that there is a rotational misalignment of angle *β* of the *u* accelerometer with respect to the *x* axis of the instrument. Given an initial sensitivity matrix with *β* = 0 that was measured in section 4.2

(35) $$\left[\begin{array}{ccc} s_{ux} & s_{uy} & s_{uz}\\ s_{vx} & s_{vy} & s_{vz}\\ s_{wx} & s_{wy} & s_{wz} \end{array}\right]=\left[\begin{array}{ccc} 0.201358 & -0.001085 & 0.000059\\ -0.002416 & 0.201607 & -0.003382\\ -0.001109 & 0.000746 & 0.202449 \end{array}\right],$$

and the sensitivity matrix as a function of rotational misalignment angle *β* is calculable as,

(36) $$\left[\begin{array}{ccc} s_{ux}(\beta ) & s_{uy}(\beta ) & s_{uz}(\beta )\\ s_{vx}(\beta ) & s_{vy}(\beta ) & s_{vz}(\beta )\\ s_{wx}(\beta ) & s_{wy}(\beta ) & s_{wz}(\beta ) \end{array}\right]=\left[\begin{array}{ccc} s_{ux}\;\cos\left(\beta \right)+s_{uy}\;\sin\left(\beta \right) & s_{ux}\;\sin\left(\beta \right)+s_{uy}\;\cos\left(\beta \right) & s_{uz}\\ s_{vx}\;\cos\left(\beta \right)-s_{vy}\;\sin\left(\beta \right) & s_{vx}\;\sin\left(\beta \right)+s_{vy}\;\cos\left(\beta \right) & s_{vz}\\ s_{wx} & s_{wy} & s_{vw} \end{array}\right].$$

Secondly, noise was added to the simulated measurements of amount *σ*_noise_ defined as the standard deviation from a time series of measurements taken at each angle of rotation during the calibration. In this work the noise was added using the NORM.INV and RAND functions in Microsoft EXCEL. The simulated responses of the (*u*, *v*, *w*) accelerometers were calculated for the (*x*, *y*, *z*) rotations using equations (9)–(11) and assuming zero offsets for the purposes of this simulation. For example, the response of the *u* accelerometer to the *x*-axis rotation angle *α* was specified as,

(37) $$U^{x}\left(\alpha_{i}\right)=\mathrm{NORM}.\mathrm{INV}(\mathrm{RAND}0,s_{uy}(\beta )\;\sin\left(\alpha_{i}\right)+s_{ux}(\beta )\;\cos\left(\alpha_{i}\right),\sigma_{\text{noise}}),$$

and similar equations were derived for the others. The simulated data were then used to calculate the intrinsic properties and the uncertainties as a function of noise and rotational misalignment.

**Figure 6.**
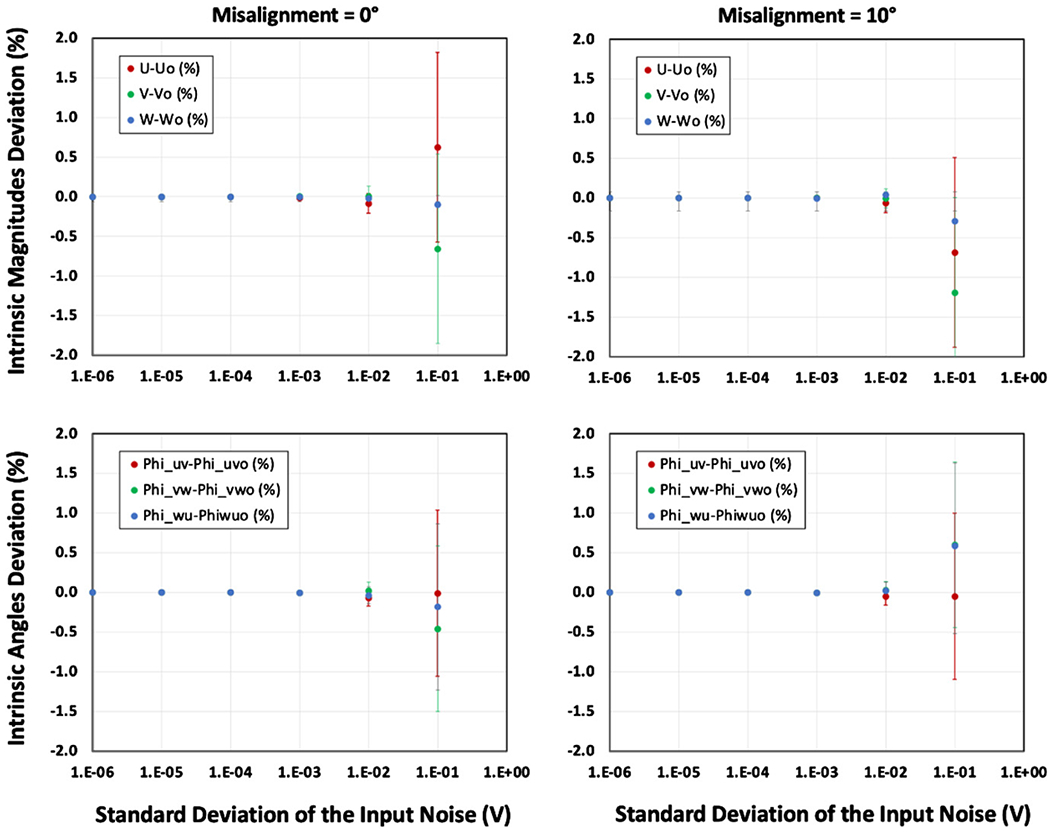
Results of the simulation for the intrinsic property of the (*U*, *V*, *W*) accelerometers and the angles between them *φ_uv_, φ_vw_, φ_vw_* as a function of logarithmically increasing σ_noise_ from 1 μV to 100 mV for 0° and 10° misalignments. The error bars exhibit the 95% confidence (*k* = 2) derived from the quality of the sine fit. The results show an expected increasing uncertainty with increasing noise yet to within the uncertainties the properties remain constant and equivalent at both 0° and 10° rotational misalignment. The intrinsic angles of deviation in (%) are with respect to the relative reference value of 90°. It should be noted that this simulation uses a random number generator to produce the results thus the values of the specific points change each time the simulation is updated but they remain equivalent to within their uncertainties.

Figure 6 shows the results of the simulation for the intrinsic property of the *U* accelerometer and the angle between the *U*–*V* accelerometers *φ_uv_* as a function of logarithmically increasing *σ*_noise_ from 1 μV to 100 mV for 0° and 10° misalignment. The results show an expected increasing uncertainty with increasing noise yet to within the determined uncertainties the properties remain constant and equivalent throughout the range of the rotational misalignment simulated of 0° and 10°.

**Figure 7.**
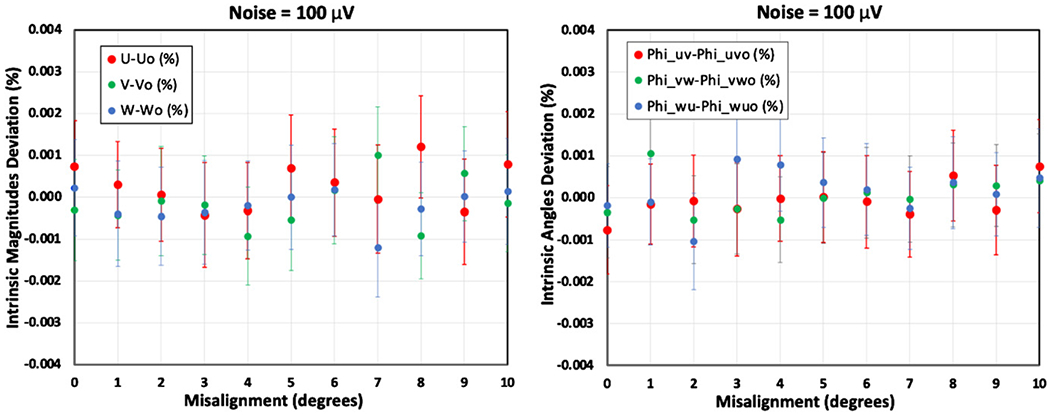
Simulation of the intrinsic properties as a function of misalignment angle with a 100 μV noise level on the accelerometer output voltage. The error bars exhibit the 95% confidence (*k* = 2) derived from the quality of the sine fit. It is observed that to within the uncertainty depicted by the error bars that the values are in agreement throughout the full range of the misalignments. The intrinsic angles of deviation in (%) are with respect to the relative reference value of 90°. It should be noted that this simulation uses a random number generator to produce the results thus the values of the specific points change each time the simulation is updated but they remain equivalent to within their uncertainties.

Figure 7 shows simulation results of the intrinsic properties as a function of increasing misalignments in 1° steps from 0° to 10° at a constant *σ*_noise_ of 100 μV. This noise level was chosen because it was slightly greater than the standard deviation of the fit to the data observed in the initial calibration reported in section 4.2. The deviation of the intrinsic properties is with respect to the 0° misalignment condition and reported as a percentage deviation with respect to its value at 0° misalignment. These results show that the model predicts that intrinsic properties for both the magnitudes and angles will remain constant to within the 0.03% (*K* = 2) deviation derived from the quality of the fit of the data at this noise level.

## 5. Experiment on the effect of misalignment

In this section we report on the results of two experiments to investigate the effects of misalignment of the DUT on the calibrated intrinsic properties and sensitivity matrix. The first experiment reports the results of a controlled misalignment, where the degree of misalignment was introduced by adding it as a constant value to the rotational position of axis 1 of the measuring instrument. The second experiment involved removing the DUT from the measuring instrument and reattaching it for each measurement repeat, introducing an unknown, quasi-random amount of misalignment.

### 5.1. Controlled misalignment

**Figure 8.**
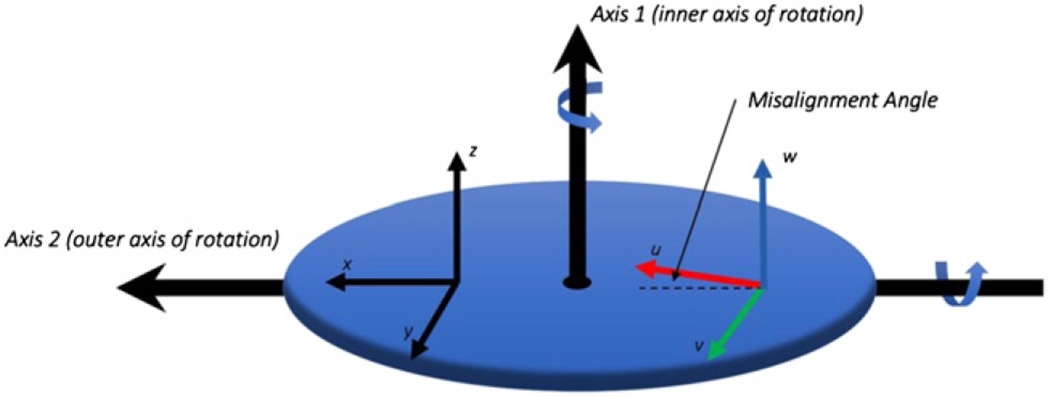
Depiction of the misalignment angle used in the experiment.

The procedure used to experimentally investigate the misalignment effect on the intrinsic properties in comparison to the sensitivity matrix in a controlled manner was essentially the same as what was described in the simulations carried out in section 4.4. A misalignment angle of *β* ∈ {−5°, −4°, … , 0°, … ,4°, 5°} was added to axis 1 of the instrument for each calibration condition as depicted in figure 8.

**Figure 9.**
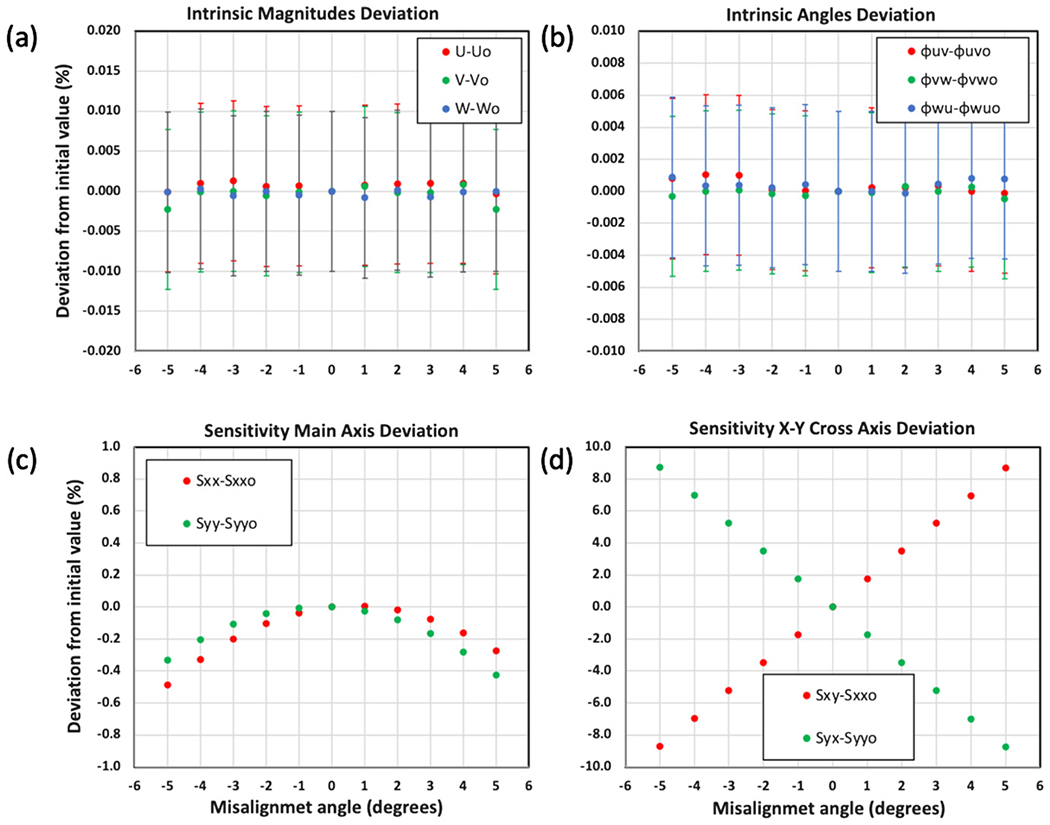
The effect of mounting misalignment angle Δ ∈ {−5°, −4°, … 0°, … 4°, 5°}. The data are color coded red, green, blue to represent the *U*, *V*, *W* accelerometers, respectively. Plot (a) shows the percent deviation from the initial calibration of the *U*, *V*, *W* accelerometers and (b) shows the intrinsic angles *φ_uv_, φ_vw_, φ_vw_* both with respect to the initial calibration with 0° misalignment. Plot (c) shows the percent deviation of the diagonal values of the sensitivity matrix *S_xx_, S_yy_, S_zz_* and (d) the cross-axis terms. The elements of the matrix related to the *Z* axis showed relatively little variation as would be expected since the misalignment was confined to rotation in the *X*–*Y* plane. The intrinsic angles of deviation in (%) are with respect to the relative reference value of 90°. The error bars exhibit the 95% confidence (*k* = 2) derived from the quality of the sine fit, which are not visible in plots (c) and (d) because of their small size with respect to the markers.

Figure 9 shows the results of the experiment. Figure 9(a) compares the deviation of the intrinsic property magnitudes *U*, *V*, and *W* to the main axis terms of the inverse sensitivity matrix i*s_xx_*, i*s_yy_*, and i*s_zz_* in figure 9(c). It is observed that both the magnitudes of the intrinsic properties remain constant within their stated uncertainties as well as their associated intrinsic angles $\varphi_{uv},\varphi_{vw},$ and $\varphi_{wu}$ shown in figure 9(b). On the other hand, the *x*–*y* main axis terms of the sensitivity matrix, *s_xx_* and *s_yy_*, show a cosine-type of relationship in their deviation, reaching a maximum deviation of order 5% at 5° misalignment. The deviation observed for the *x–y* terms of the sensitivity terms, *s_xy_* and *s_yx_*, exhibit a more strongly related variation as a function of the misalignment angle approaching 10% at 5° misalignment. This indicates that uncertainty of these inverse sensitivity terms would dominate down to 0.01° misalignment.

The *z* axis main term *s_zz_*, as well as the other *z*-axis related inverse sensitivity components *s_xz_*, *s_yz_*, *s_zx_*, and *s_zy_* show relatively little relation to the misalignment as would be expected since the misalignment was confined to the *x–y* plane. The uncertainties related to the deviation of the sensitivity matrix values are small compared to their observed variation, on the order of 0.01%, making their error bars smaller than the markers drawn in the plots.

### 5.2. Misalignment introduced by remounting the DUT

**Figure 10.**
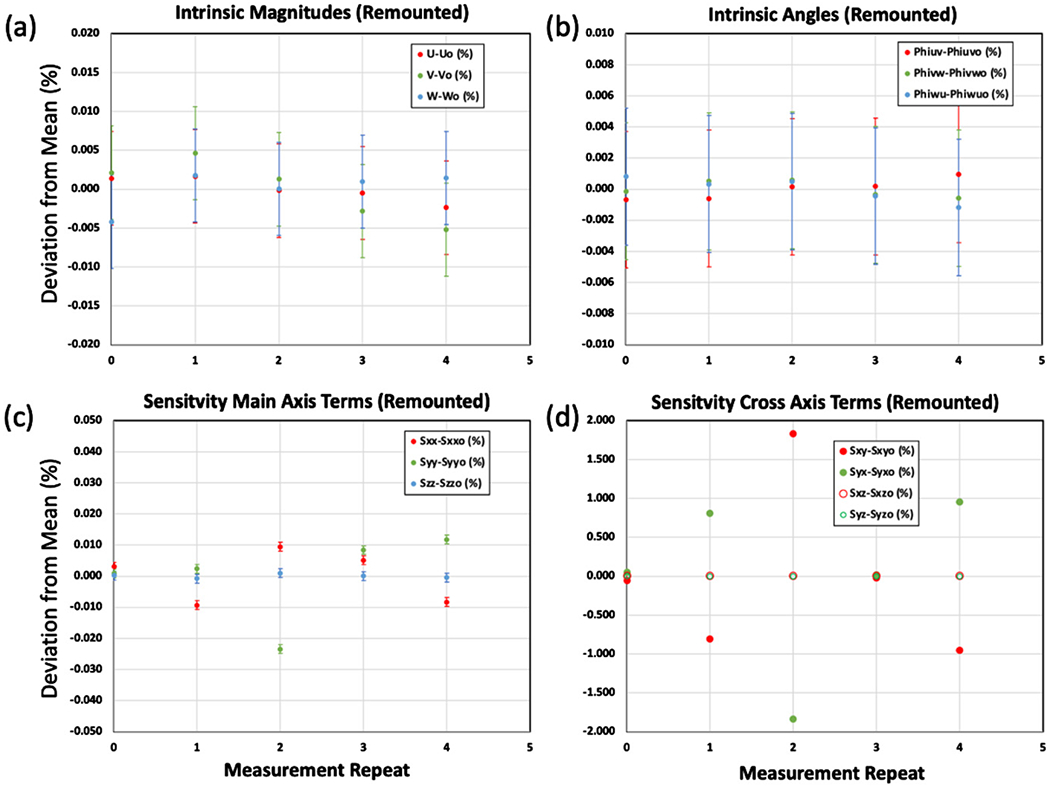
Results for measurement repeats where the DUT was removed and remounted onto the measurement apparatus for each repeat. Plots (a) and (b) show the deviation from the mean of the sensitivity of the *U*, *V*, *W* accelerometers and the intrinsic angles *φ_uv_, φ_vw_, φ_vw_* respectively. Plots (c) and (d) show the deviation from the mean for the main axis terms of the sensitivity matrix *S_xx_, S_yy_, S_zz_* and the cross-axis terms *S_xy_, S_yx_, S_xz_, S_yz_*, respectively. The error bars can be seen in (c) since the variation is small enough to depict them however in (d) the variation is too large to depict them. The intrinsic angles of deviation in (%) are with respect to the relative reference value of 90°. It is observed that the intrinsic properties remain constant to within the (*K* = 2) uncertainties due to the degree of the sine fit but the sensitivity matrix terms do not. Therefore, the use of the sensitivity matrix for laboratory comparisons or measurement repeats where the DUT is removed for each repeat will require an additional uncertainty component due to misalignment whereas the use of intrinsic properties do not.

In this experiment the DUT was removed and remounted onto the measurement instrument for each measurement repeat which can be thought of as introducing an uncontrolled amount of misalignment to within the amount of play in the mounting apparatus. The calibration was repeated for five mounting conditions and the results are plotted in figure 10. It is observed that the intrinsic properties remain constant to within the (*K* = 2) uncertainties attributed by the residuals of the sine fit but the sensitivity matrix terms do not. These results demonstrate that the use of the sensitivity matrix for laboratory comparisons or measurement repeats will require an additional uncertainty component due to misalignment whereas the effect of the misalignment is alleviated by the use of intrinsic properties.

For example, suppose that 10 calibration laboratories measure the sensitivity matrix and intrinsic properties of the same tri-axis accelerometer. Furthermore, suppose that the standard deviations of the sensitivity matrix between laboratories are much larger than those of the intrinsic properties, then the former can be attributed almost entirely to differences in the alignments of the accelerometer in each laboratory. On the other hand, if the standard deviations of the sensitivity matrix between laboratories are comparable to the standard deviations of the intrinsic properties, then the former can be attributed almost entirely due to errors the measurement instruments such as angular rotation error or non-orthogonality of the rotational axes.

## 6. Conclusion

We have presented a detailed analysis and application of the use of intrinsic properties for tri-axis accelerometer calibration based on rotation of the DUT in the gravitational field. The experimental protocol and analysis were simulated to include the effects of measurement noise and misalignment angle. The simulations showed an increasing uncertainty with increasing noise yet to within their uncertainties the intrinsic properties remained constant and equivalent throughout the noise range investigated up to 100 mV and a rotational misalignment investigated up to 10°. We then showed experimentally that the use of the intrinsic properties for tri-axis accelerometer calibrations alleviates uncertainties due to misalignment of the DUT using a controlled misalignment condition and by measurement repeats where the DUT was remounted with each measurement repeat. These sorts of conditions are of particular importance for comparisons between calibration laboratories where attaining the lowest possible uncertainties are of interest. Our results indicate that the uncertainties of the cross-axis terms of the sensitivity matrix are dominated by the misalignment angle down to a 0.01° at 100 μV noise. These uncertainties would dominate at even smaller angles of misalignment if the measurement noise were further reduced. We conclude that the use of intrinsic properties will yield lower uncertainties for laboratory comparisons and for measurement repeats where the DUT is remounted for each calibration.

## Figures and Tables

**Table 1.** Values in the units of volts for variables *A, B*, and *O* determined by sine fitting using the LINEST function in Microsoft Excel for the measurements shown in figures 4 and 5.

(a) Rotation around *X*			
0	$A_{uy}^{x}:$ −0.010 809	$B_{uz}^{x}:$ 0.000 919	$O_{u}^{x}:$ −0.005 59
0	$A_{vy}^{x}:$1.975 968	$B_{vz}^{x}:$ −0.032913	$O_{v}^{x}:$ 0.014132
0	$A_{wy}^{x}:$ 0.007 058	$B_{wz}^{x}:$ 1.984 217	$O_{w}^{x}:$ 0.002 869
(b) Rotation around *Y*			
$A_{ux}^{y}:$ 1.973 531	0	$B_{uz}^{y}:$ 0.000 244	$O_{u}^{y}:$ −0.004931
$A_{vx}^{y}:$ −0.023 513	0	$B_{vz}^{y}:$ −0.033 380	$O_{v}^{y}:$ 0.013 402
$A_{wx}^{y}:$: −0.010 639	0	$B_{wz}^{y}:$ 1.984 207	$O_{w}^{y}:$ 0.002 850
(c) Rotation around *Z*			
$A_{ux}^{z}:$ 1.973 499	$B_{uy}^{z}:$−0.010 457	0	$O_{u}^{z}:$ −0.004913
$A_{vx}^{z}:$ −0.023 842	$B_{vy}^{z}:$1.975 928	0	$O_{v}^{z}:$0.014103
$A_{wx}^{z}:$−0.011098	$B_{wy}^{z}:$0.007 554	0	$O_{w}^{z}:$ 0.003 180

## Footnotes

3 There is discrepancy in the industry concerning the terminologies of sensitivity. By definition, sensitivity measures the output gain as a function of input of a detector system. For the case of an accelerometer, sensitivity measures the electrical output per acceleration input and its units should be specified as V (m^−1^ s^−2^). However, some industry segments regularly use the units of (V/*g_n_*) for sensitivity and other segments use the term (*g*_*n*_/LSB) [10]. The datasheet for the device used in this work lists 2000 mV/*g*_*n*_ as a specification for its sensitivity.
